# Households with unimproved water sources in Ethiopia: spatial variation and point-of-use treatment based on 2016 Demographic and Health Survey

**DOI:** 10.1186/s12199-020-00921-1

**Published:** 2020-12-07

**Authors:** Yohannes Tefera Damtew, Abraham Geremew

**Affiliations:** Department of Environmental Health, College of Health and Medical Sciences, Haramaya University, Dire Dawa, Ethiopia

**Keywords:** Unimproved water, Spatial variation, Local clusters, Point-of-use treatment

## Abstract

**Background:**

Improved water sources are not equally available in all geographical regions. Populations dependent on unsafe water sources are recommended to treat their water at point-of-use using adequate methods to reduce associated health problems. In Ethiopia, the spatial distribution of households using unimproved water sources have been incomplete or ignored in most of the studies. Moreover, evidence on the point-of-use water treatment practice of households dependent on such water sources is scarce. Therefore, the current study is intended to analyze the spatial distribution of unimproved water sources by wealth quintiles at country level and point-of-use treatment (POU) practices using nationally representative data.

**Method:**

The data of 2016 Ethiopian Demographic and Health Survey (EDHS) conducted on 16650 households from 643 clusters were used for the analysis. For spatial analysis, the raw and spatially smoothed coverage data was joined to the geographic coordinates based on EDHS cluster identification code. Global spatial autocorrelation was performed to analyze whether the pattern of unimproved water coverage is clustered, dispersed, or random across the study areas. Once a positive global autocorrelation was confirmed, a local spatial autocorrelation analysis was applied to detect local clusters. The POU water treatment is analyzed based on reported use of either boiling, chlorine (bleach), filtration, or solar disinfection (SODIS).

**Results:**

There were 5005 households using unimproved water sources for drinking purposes. Spatial variation of unimproved water coverage was observed with high coverage observed at Amhara, Afar, Southern Nations Nationalities and People and Somalia regions. Disparity in unimproved water coverage between wealth quintiles was also observed. The reported point-of-use water treatment practice among these households is only 6.24%. The odds of POU water treatment among household heads with higher education status is 2.5 times higher (95% CI = 1.43-4.36) compared to those who did not attend education.

**Conclusion:**

An apparent clustering trend with high unimproved water coverage was observed between regions and among wealth quintiles hence indicates priority areas for future resource allocation and the need for regional and national policies to address the issue. Promoting households to treat water prior to drinking is essential to reduce health problems.

## Background

Access to improved water is a fundamental human right [1]. However, it is not equally available in all geographical regions (He et al. [2]). A series of studies have shown that location and socio-economic status are the most pronounced factors for unequal access to improved drinking water [3, 4]. This is clearly seen, such as, in the analysis of five African and Latin American countries which showed a clear pattern of inequality for broad regional units and more at local scale and different wealth classes [5]. Hence, the population dependent on such water suffers a lot from different health problems of which diarrhea is one [6]. On the other hand, populations dependent on unsafe water sources are strongly recommended to treat their water at point-of-use (POU) to reduce health problems associated with gastrointestinal infections (e.g., diarrhea), even if it is not routinely and widely practiced [7–9].

In Ethiopia, according to WHO/UNICEF 2015 progress report on sanitation and drinking water, 43% of populations depend on unimproved water sources [10]. Previous studies [11–14] indicated the association between diarrhea, contaminated water sources, and effectiveness of POU water treatment. In addition, the households’ water treatment practices using SODIS, chlorine, boiling, and filtration at the national level are acknowledged [15, 16].

But, evidence on the spatial distribution of households which depend on unimproved water sources has been incomplete or ignored in most of the studies [2, 17]. In this regard, few studies were conducted in Ethiopia to locate or map areas with highest unimproved water source coverage among different wealth categories and local levels [18, 19]. Moreover, data on how POU water treatment practices of populations dependent on unimproved water sources is scarce. Therefore, this could have influence on intervention measures and makes it impossible to trace sources of water-borne epidemics [20, 21].

Thus, the main objective of this study is to analyze and map the spatial distribution of unimproved water coverage by wealth quintile in detail and demonstrate disparities in unimproved water coverage not only across regions but also within regions between wealth quintiles. In addition, it was intended to assess the POU water treatment practices of households dependent on such water sources. The finding will aid policymakers, partners, and planners, in the water and health sector to develop appropriate strategies in improving water source development, quality monitoring, and measures like wide-scale use of household water treatment methods [22–24]. It will also be helpful to ensure the United Nations sustainable development goal (SDGs), ensuring availability and sustainable management of water for all. The goal reflects the increased ambition for improving access to the unserved and reducing the inequality in improved drinking water supply [25].

## Method

### Study setting

The world population review indicates that Ethiopia has an estimated population of 114.96 million in 2020 that makes the second populous country in Africa [26]. The country has an administrative structure of nine regional states (Tigray, Afar, Amhara, Oromiya, Somali, Benishangul-Gumuz, Southern Nations Nationalities and People Region (SNNPR), Gambela, and Harari) and two city administrations (Addis Ababa and Dire Dawa) [27]. A finding on diarrhea and associated factors in the country revealed that the pooled prevalence of diarrhea among under-five children was 22%. The analysis of subgroup in this study showed that the highest prevalence was observed in the Afar region (27%), followed by the Somali and Dire Dawa regions (26%), then Addis Abeba (24%) [28]. Studies on the prevalence of childhood diarrhea with the type of water source they use show that high prevalence was observed on those households with unimproved drinking water sources [29, 30]. On the other hand, less than half (41%) of the total populations have at least basic drinking water services level, 28% have limited services (more than 30 min), 22% have unimproved services, and 9% use surface water as a source of drinking water supply in the country [31].

### Study design and data source

The EDHS data collected in 2016 was used. We obtained the data through online registration on MEASURE DHS program. A two-stage stratified sampling design based on nationally representative household surveys was implemented. Although EDHS has different data files, the household (HR) data file was used in this study.

### Data processing

In total, 16,650 households from 643 clusters are included and each cluster was represented by a GPS point with latitude and longitude coordinates. After initial data processing, 22 clusters without GPS points were excluded. For the final data analysis, 621 clusters and 5005 (weighted frequency of 5857) were identified to be using water from unimproved water sources based on the WHO/UNICEF category [32]. These households were used to analyze POU household water treatment practice. GPS coordinate displacement was performed on the actual locations of each cluster to produce data with displaced distances to maintain confidentiality of the surveyed respondents [33]. For urban clusters, a displacement toward a random direction by a maximum of 2 km was implemented. Rural clusters were displaced to a maximum of 5 km, with a further 1% displaced to a maximum of 10 km. A subset of clusters with GPS points were created for each wealth quintile. Since a single cluster was represented by a single GPS point and households in a single DHS cluster may fall in different quintiles, subsets of clusters were not mutually exclusive.

### Data analysis

We have constructed a new wealth quintile by excluding drinking water as an asset of the households. The default asset scores and quintiles of DHS datasets were constructed by including drinking water supply as an input. However, drinking water is the dependant variable of this study and was excluded to construct wealth quintiles. An asset score for each household was constructed using Principal Components Analysis in Stata 14.0 [34]. All surveyed households were ranked and divided into five subsets or wealth quintiles. The first quintile included the poorest 20% of households and the fifth quintile included the wealthiest 20%. Following the approach used by Jia et al. [35] five subsets of clusters with GPS points representing five quintiles were created. Each subset included all the clusters that contained at least one household in the corresponding quintile. Since a single cluster was represented by a single GPS point and households in a single DHS cluster may fall in different quintiles, subsets of clusters were not mutually exclusive.

The raw coverage rate of unimproved water in each cluster was calculated as a proportion. The proportion was calculated as households with any of unimproved water sources to the total households in each cluster for the overall population and for each quintile. It has also accounted for the survey design and weight. The difference in raw coverage rates among the sampled clusters was statistically tested using one-way ANOVA.

A spatially smoothed rate was calculated to stabilize raw rates. To perform the smoothing, first, a Thiessen polygon which divides an area into regular sub-areas that encloses all locations closer to the central point than to any other point was created [36]. Spatial smoothing was used to produce a corresponding estimate to the raw coverage rate of each cluster from a collection of neighboring clusters enclosed by Thiessen polygon. For this study, the first order Queen Contiguity was applied as the spatial smoothing rule. Queen Contiguity spatial smoothing rule considers all neighboring polygons sharing a common edge or a common vertex with the target Thiessen polygon as neighbors. The difference between spatially smoothed and raw coverage rates for the overall population and each quintile was also calculated by subtracting the raw coverage from spatially smoothed coverage rates.

Spatial autocorrelation was performed by joining the raw and spatially smoothed coverage data to the geographic coordinates based on DHS cluster identification code. We have assumed there is a complete randomness of unimproved water distribution in the study sites. Global spatial autocorrelation was performed to analyze whether the pattern of unimproved water coverage is clustered, dispersed, or random across the study areas. The Global Moran’s *I* measure spatial autocorrelation based on the feature locations and attribute values. For a set of features with associated attribute, Global Moran’s *I* evaluate whether the pattern expressed is clustered, dispersed, or random. When the *z* score or *p* value indicates statistical significance, a positive Moran’s *I* index value indicates tendency toward clustering while a negative Moran’s *I* index value indicates tendency toward dispersion. As the global spatial autocorrelation technique provides one quantitative value for the whole dataset, it cannot identify local clusters with high or low coverage. Thus, local spatial autocorrelation analysis was applied to detect local clusters for positive global autocorrelation results. Local Moran’s *I* was used to calculate a test statistic for each location and to identify clusters of high and low coverage. A random permutation procedure (RPP) was used to replicate the statistics 999 times to generate reference distributions. The distribution of the test statistics was evaluated against a theoretical or random reference distribution generated. Local Moran’s *I* was calculated for both raw and spatially smoothed rates. Both the global and local spatial autocorrelation was calculated using GeoDa [37]. For POU water treatment, the number of households reportedly use adequate water treatment methods (chlorination, boiling, filtration, and SODIS) were considered a yes (1) and no otherwise (0 = if the household had used neither of them, i.e., households which had used either let it stand and settle, cloth straining, or never used any treatment option). Descriptive and logistic regression was used to assess the associated factors with the household POU treatment. A multivariable logistic regression was run to identify factors associated with POU water treatment practices by including variables with *p* value < 0.25 from bivariate analysis.

## Results

### Raw and spatially smoothed coverage

**Fig. 1:**
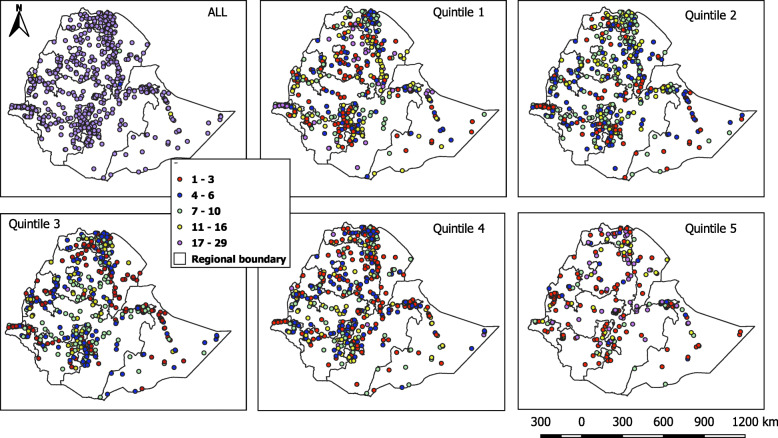
Number of households in each cluster for the overall population and each quintile. There are 621 total clusters included in this study after initial data processing, with 601 clusters including 20–29 households and the remaining 20 clusters including 5–19 households for the overall population. Each of the five quintiles included all the clusters that contained at least one household with an asset score in the corresponding quintile. The number of households in each cluster ranges between 1-29 per cluster

Five subsequent wealth quintiles or subsets of clusters have been created based on the national assets score, excluding drinking water supply. Accordingly, households were assigned to one of the five wealth quintiles. Clusters categorized by the number of households each cluster denotes for each quintile are presented in Fig. 1. The number of households varies across quintiles with 26.9-41.2% of clusters had only 1-3 households, meanwhile, 11.3-48.2% of the clusters possessed 17-29 households. The spatial distribution of clusters by quintile and for the overall population (Fig. 1) showed a similar distribution of clusters in the first three quintiles, while quintile four and five had a greater number of clusters around the capital Addis Ababa. The first three quintiles had a higher number of clusters at the North (Tigray and Amhara regions) and South (SNNPR). The trend continued to the fourth quintile, except the distribution of clusters extended to the center and around the capital, Addis, Ababa. The fifth quintile had a similar pattern around the capital and clusters sparsely distributed at the periphery.

**Fig. 2:**
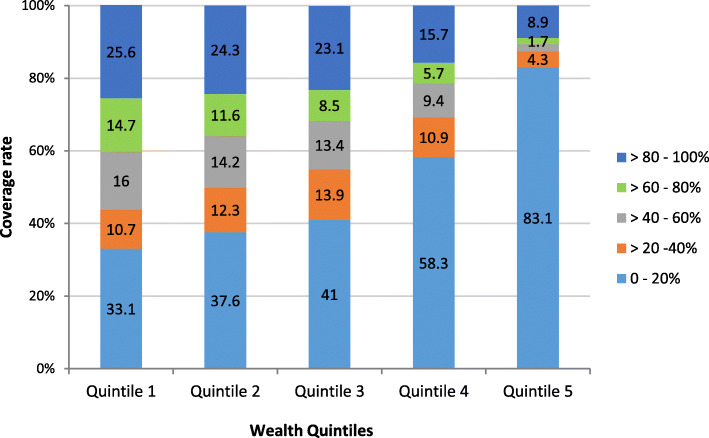
Numbers and percentages of clusters categorized by the raw coverage rates of unimproved water by quintile. The percentage of clusters with a high number of unimproved water coverage (80-100%) declined from the poorest to the richest quintile, while clusters with 0-20% coverage rise in the same direction

**Fig. 3:**
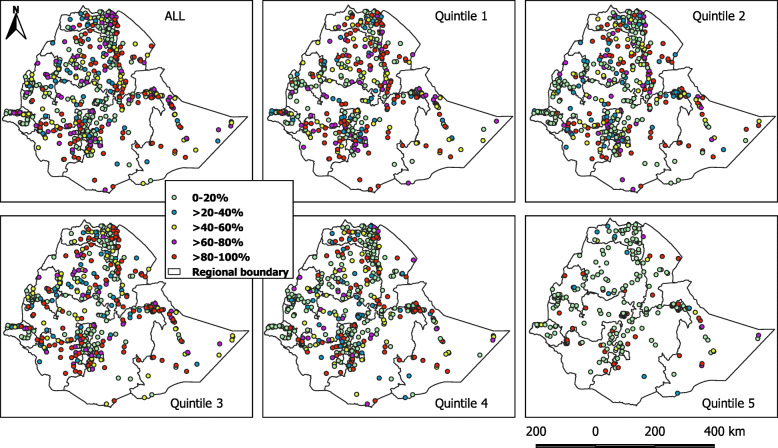
Clusters categorized by the raw coverage rates of unimproved water for the overall population and by each quintile. Clusters with a higher percentage of unimproved water coverage were located at the North, South, and East part of the country. The red and pink colors indicate clusters with the highest percentage of unimproved water coverage

The number of clusters with varying percentage of raw coverage by wealth quintile is presented in Fig. 2. The percentage of clusters with a high number of unimproved water coverage (40-100%) declined from the poorest quintile (56.3%) to the richest (12.6%). The difference in raw coverage rates among the sampled clusters was statistically tested using one-way ANOVA. The difference in raw coverage between quintiles was statistically significant, *F* (3, 1697) = 21.1, *p* < 0.001. High percentage of unimproved water coverage was observed in the first three quintiles compared to quintile four and five which had a small number of households with unimproved water source. Spatially, clusters with a higher percentage of unimproved water coverage were located at the North (Amhara and Afar regions), South (SNNPR), and East (Somalia) regions (Fig. 3). While in the capital Addis Ababa, Dire Dawa City administration, and Gambella region, the coverage of unimproved water is low.

**Fig. 4:**
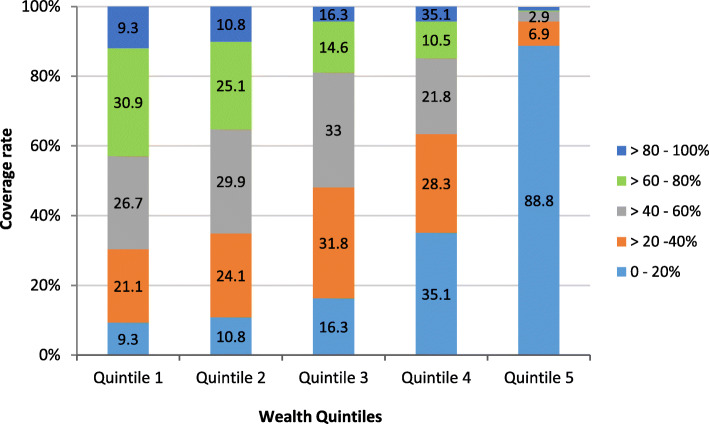
Numbers and percentages of clusters categorized by the spatially smoothed coverage rates of unimproved water. The percentage of clusters with 0-20% of unimproved water coverage declined, while the percentage of clusters with 60-80% rise in the first four quintiles after the spatial smoothing process

**Fig. 5:**
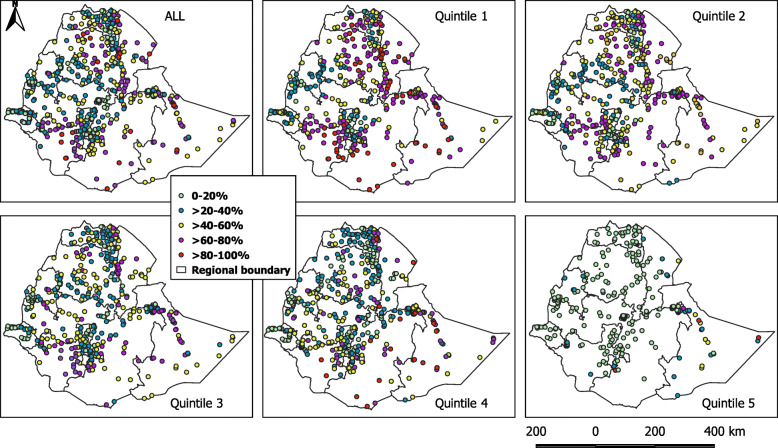
Clusters categorized by the spatially smoothed coverage rates of unimproved water for the overall population and by each quintile. The percentage of unimproved water coverage was high in the North, South, and East with a clear pattern after the spatial smoothing process

As 26.9-41.9% of the clusters in different quintiles had only three or less households, a spatially smoothed coverage rate was calculated for each cluster to overcome the small number issue and to identify the cluster trend better. Figure 4 showed clusters categorized by smoothed coverage in each quintile, while the distribution of spatially smoothed coverage rate for all populations and by quintile is presented in Fig. 5. The percentages of clusters with 0-20% and > 80-100% coverage has decreased in all quintiles except quintile 5, which showed a rise in the percentage of clusters with 0-20% coverage. The pattern of clusters with > 60-100% coverage of unimproved water was clearly observed at the North, South, and East parts of the country and becomes infrequent as we moved from the first quintile to the fifth.

The difference between spatially smoothed and raw coverage rates for the overall population and each quintile was calculated by subtracting the raw coverage from spatially smoothed coverage rates. Clusters with a small number of households showed a considerable change by the spatial smoothing process.

### Hotspots and clustering trends

**Fig. 6:**
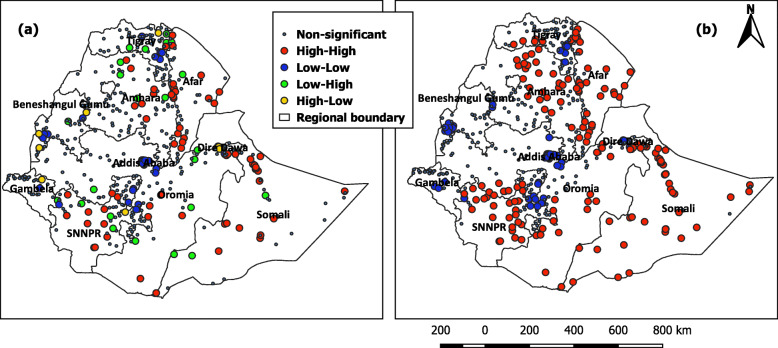
Clustering trends of coverage rates before (**a**) and after spatial smoothing (**b**). Clustering of spatial outliers was eliminated in the spatially smoothed coverage. The red colors indicate clusters with the highest percentage of unimproved water while the blue colors show clusters with low unimproved water coverage

The global spatial autocorrelation was calculated using Global Moran’s *I*. The analysis based on feature locations and attribute values revealed a clustering pattern of unimproved water coverage across the whole clusters (Global Moran’s *I* = 0.174, *p* value < 0.0001). Following a statistically significant positive result from the global spatial autocorrelation, Local Moran’s *I* was also calculated for the overall population to show hot and cold spots. Unlike the global spatial autocorrelation, local spatial autocorrelation was calculated for both the raw and spatially smoothed data. The local clustering trend of high and low spots among sampled clusters was identified in both raw and spatially smoothed coverage (Fig. 6a and b). However, the clustering of spatial outliers was eliminated in the spatially smoothed coverage. The raw coverage revealed 72 high-high and 120 low-low statistically significant clusters (*p* < 0.05), followed by 8 high-low and 27 low-high clusters. Meanwhile, the spatially smoothed coverage showed 155 high-high and 149 low-low statistically significant clusters (*p* < 0.05). Clusters in the North (Amhara region and Afar), in the East (Somalia region), and in the south (SNNPR region) had statistically significant unimproved hot spots (95% confidence). Clusters in the center, South, and West had statistically significant cold spots as presented in Fig. 6a and b.

### Point-of-use treatment practices

Of the total households (5005), 613 (10.46%) treat their water prior to drinking with any of the methods (use either boiling, filtration, chlorination, SODIS, let it stand and settle, and cloth straining) and 365 (6.24%) of households treat using adequate methods (use either boiling, filtration, chlorination, or SODIS). The number of households and reportedly used treatment methods are boiling 125 (2.14%), chlorination 164 (2.80%), cloth straining 204 (3.48%), filtration 105 (1.80%), let it stand and settle 41 (0.70), and SODIS 5 (0.09%). The data shows that of the adequate methods, treating with SODIS the least to be used by the households in the country (Table 1).

**Table 1:** Point-of-use water treatment practices among households with unimproved water sources and associated factors, EDHS, 2016

Factors	Category	***N*** (%)	POUWT		AOR (95% CI)
			No	Yes	
Owning Radio	No	4,671 (79.76)	4393	279	
	Yes	1,185 (20.24)	1099	87	1.25 (0.96, 1.64)
Owning Television	No	5,817 (99.33)	5458	360	
	Yes	34 (0.67)	34	87	0.52 (0.24, 1.13)
Household wealth quintile	Poorest	1,956 (33.40)	1825	132	
	Poorer	1,533 (26.18)	1461	73	0.58 (0.43, 0.79)
	Middle	1,355 (23.14)	1289	66	0.71 (0.52, 0.97)
	Richer	911 (15.55)	841	70	0.80 (0.57, 1.11)
	Richest	101 (1.73)	75	26	1.97 (1.12, 3.47)
Household head education	No education	3,805 (65.22)	3605	200	
	Primary	1,765 (30.26)	1633	132	1.31 (1.02, 1.68)
	Secondary	179 (3.06)	161	18	1.33 (0.79, 2.22)
	Higher	85 (1.46)	71	15	2.50 (1.43, 4.36)
Residency	Rural	91 (1.55)	5412	354	
	Urban	5,766 (98.45)	80	11	0.91 (0.52, 1.61)
Presence of Children in the house	No	2,445 (41.75)	2288	157	
	Yes	3111 (58.25)	3203	209	1.02 (0.82, 1.27)

*AOR* adjusted odds ratio, *CI* confidence interval, *POUWT* point-of-use water treatment

The logistic regression shows that the odds of treating water among household heads with the highest education level was 2.50 more (95% CI = 1.43, 4.36) compared to those who did not attend formal education. Household with the highest wealth quintile had more odds of treating water compared to the poorest (Table 1).

## Discussion

Narrowing the gap in service inequality, particularly access to improved water as a human right is viewed as a significant post-Millennium Development Goals actions [2, 5, 21]. The current study analyzed the spatial distribution of unimproved water coverage in Ethiopia by wealth categories. Households included in the first three quintiles were majorly located in the Northern and Southern parts of the country. Simultaneously, these are regions with the highest percentage of unimproved water coverage, with additional clusters in the east (Somalia region). Meanwhile, the last two wealth categories had more households around the capital compared to the first three wealth quintiles. The percentages of households with low unimproved water coverage were linked with the latter two quintiles. Studies indicated that socio-economic status can be a critical factor to access improved drinking water [38, 39]. The findings in this study also show hotspots of unimproved drinking water sources at the national and regional levels. Most of the clusters were in pastoral and rural areas of Afar, on the border of Amhara and Afar, Somalia, and SNNPR regions. Pastoralists are among the most poorly served population in Ethiopia, a country that has low levels of water access. This can be due to low priority for rural areas and pastoral communities. Moreover, water sources in these areas are few and far between, which could result contamination of water between point of collection and consumption [16, 40]. These rural areas and pastoral communities are also the most affected groups from diarrheal disease and related water-borne infections [14, 41]. The current findings related to unimproved coverage in these regions were indicated in a national survey [27]. The spatial distribution of unimproved water sources observed among regions and wealth quintiles was similar with previous studies [18, 42]. It also corroborates studies, which indicated variations in access to improved water attributed by wealth status [2, 17]. In another spatial analysis perspective, the high coverage of unimproved water at the North and South could be partly attributed to the large number of clusters sampled compared to the small number of clusters in the East and the center [27].

Local Moran’s *I* clustering trend of unimproved water coverage for each cluster to neighboring clusters proved presence of spatial variation.

A clear clustering trend indicating areas of high and low raw coverage surrounded by neighboring clusters with matching raw coverage rates were observed. In contrast, high coverage clusters surrounded by low coverage clusters and vice versa were sporadically observed across the country. A spatial smoothing was applied to discern systematic patterns in the spatial variation of coverage rates in such areas [14, 43]. Such clustering trends within regions could be due to rural areas surrounding urban areas at a closer proximity. This could be an indicator of inequality in access to drinking water in urban and rural areas. This study could help regional and national governments and stakeholders for urban-rural integrated water supply scheme [44, 45].

In regions with large differences in coverage before and after smoothing, further surveys and analytical methods are needed to confirm the representativeness of surveyed clusters. This was specifically an issue when we look at the spatial patterns of coverage where most of the surveyed households in rural North and South of Ethiopian categorized in the first three quintiles, while all surveyed households in Addis Ababa and Dire Dawa were in urban areas and assigned in the last two quintiles [27].

Of the total households using unimproved water sources, only 6% reportedly treat their water using adequate methods despite the finding that shows population dependent on such water sources are at risk of different health problems [6]. The point-of-use water treatment of the households association with education status of household head and wealth quintile comply with prior findings among all households included in the survey in the country [15], and specific study conducted in a specific part of the country [46], and other countries [47, 48].

In total, most households dependent on unimproved water sources are rural dwellers and a small number of households treat water prior to drinking. This could be related to low perception about the water quality and associated health risks [47, 49, 50] and the health benefits of treating water prior to treating [51–53]. The number of households reportedly using different type of treatment methods is almost comparable except SODIS which is the lowest compared to other methods. The point-of-use water treatment in general is very low despite the government reports that show effort to improve the coverage [54, 55]. As the prevalence of childhood diarrhea among households dependent on unimproved water sources is high [29, 30], ensuring wide-scale use of the POU water treatment options on these populations is fundamental for its reduction [13, 56].

## Limitations of the study

The samples taken from some regions like Somalia region was small compared to other regions. This may influence the spatial smoothing process due to the small number issue. Displacement of locations was performed to ensure confidentiality of respondents. Thus, the location of clusters may not be precise and exact. Nevertheless, the displacement was only within the administrative regions and will not influence clustering trends among regions. Household water treatment assessment was based on respondents self-report and there was no confirmatory testing of treated water.

## Conclusion

The study revealed spatial variation in unimproved water coverage across regions. Statistically significant local clusters with high coverage of unimproved water were detected in Amhara, Afar, SNNPR, and Somalia regions. Results also showed inequality between wealth quintiles within regions which may indicate the need for regional-level policy and planning to combat inequity issues. Disparity in unimproved water coverage at the spatial level and among wealth quintiles indicates priority areas for future resource allocation. Inequalities between wealth quintiles within regions may indicate the need for regional-level policy and planning in addition to national-level policies. The spatial variation and inequality in coverage of unimproved water should be dealt to address basic human rights to improved water access and to achieve sustainable development goals. This study also demonstrates the possibility and potential of spatial analysis techniques to detect inequalities in access to improved water at the regional and national levels. Household head education and wealth quintile were also statistically significant with point-of-use water treatment suggesting the need for appropriate measures for wide-scale use of the treatment methods.

## Data Availability

The datasets used and/or analyzed during the current study belong to the DHS program. The authors can provide in discussion with the data owner.

## Abbreviations

AOR: Adjusted odds ratio
DHS: Demographic Health Survey
CSA: Central Statistical Agency
FMOH: Federal Ministry of Health
EEA: Ethiopian Economic Association
GPS: Global Positioning System
SODIS: Solar disinfection
POUWT: Point-of-use treatment practices
LSMS: Living Standards Measurement Study
MoWIE: Ministry of water, irrigation and electricity
WB: World Bank
UNICEF: United Nations Children’s Emergency Fund
WHO: World Health Organization
JMP: WHO/UNICEF Joint Monitoring Programme for Water Supply, Sanitation and Hygiene
